# Long-term monitoring for conservation: closing the distribution gap of *Arctocephalus australis* in central Chile

**DOI:** 10.1186/s13104-021-05583-y

**Published:** 2021-05-06

**Authors:** Daniel Cárcamo, Marlene Pizarro, Muriel Orellana, Anahi Canto, Pablo Herrera, Lily Muñoz, Piera Vásquez-Calderón, Alicia Guerrero, Maritza Sepúlveda, L. René Durán, Doris Oliva

**Affiliations:** 1 Instituto de Biología, Facultad de Ciencias, Universidad de Valparaíso, Av. Gran Bretaña 1111, Playa Ancha, Valparaiso, Chile; 2 Centro de Investigación y Gestión de Recursos Naturales (CIGREN), Facultad de Ciencias, Universidad de Valparaíso, Av. Gran Bretaña 1111, Playa Ancha, Valparaiso, Chile; 3 Núcleo Milenio INVASAL, Concepción, Chile

**Keywords:** Population abundance, Distribution shift, South American fur seal, Peruvian fur seal, New breeding grounds, Southeastern Pacific, Humboldt Current System

## Abstract

**Objectives:**

Here, we present the first record of stable colonies of the South American fur seal (*Arctocephalus australis*), in an area where their presence has never been documented (hereafter distribution gap), as well as an update of the current distribution range of the species in central Chile.

**Results:**

A national synoptic aerial census of pinnipeds was performed during the austral summer of 2019 on the Chilean coast. An additional aerial census was conducted in the same area during the austral spring of 2019 as well as a maritime census during the austral summer of 2020. The data showed the presence of South American fur seals in central Chile within their well-known distribution gap. The total abundance was registered in three colonies where fur seals were sighted: one non-breeding colony, Punta Topocalma (summer 2019: mean = 46 ± 3; spring 2019: mean = 9 ± 1); and two breeding colonies, Punta Curaumilla (summer 2019: mean = 595 ± 7; spring 2019: mean = 45 ± 4; summer 2020: mean = 744 ± 5) and Isla Santa María (summer 2019: mean = 246 ± 6). Specifically, we suggest that it is crucial to elucidate the origin of the described settled colonies, and to determine whether there has been an augment in the distribution range from either the northern population, the southern population, or both simultaneously.

**Supplementary Information:**

The online version contains supplementary material available at 10.1186/s13104-021-05583-y.

## Introduction

**Fig. 1:**
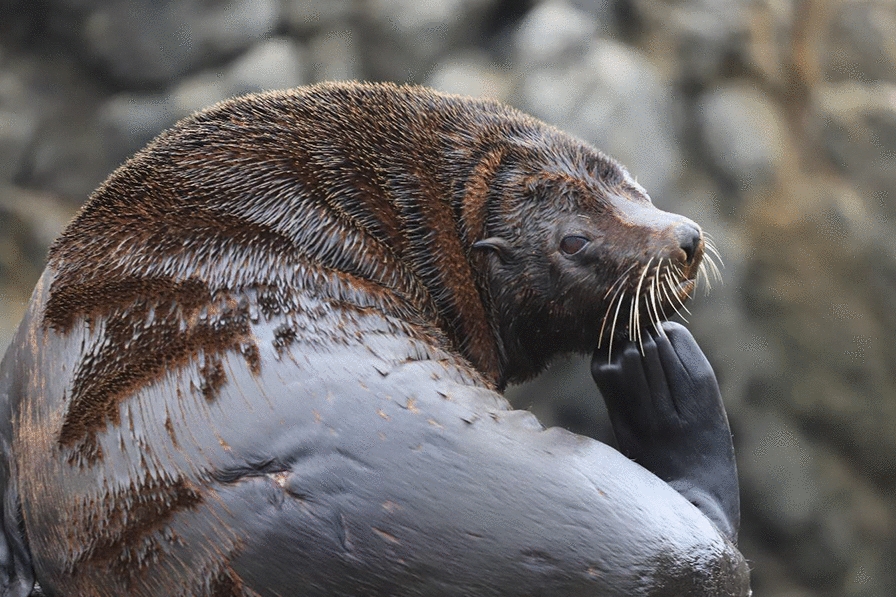
South American fur seal (*Arctocephalus australis*) sighted at the Punta Curaumilla breeding colony on March 02, 2020. Photo credit: L. René Durán. Image freely available to use

The South American fur seal (*Arctocephalus australis*, hereafter SAFS) (Fig. 1) is distributed along the Atlantic and Pacific coasts of South America [1]. The species has an estimated abundance of 219,000 individuals [2]. The distribution in the southeastern Pacific is discontinuous, with a 1600 km gap that extends from 29° 02′ S to 43° 36′ S along the coast of Chile [3, 4]. The Peruvian/northern Chile and the southern Chile/Atlantic populations of SAFS have been classified as different evolutionary units or even a subspecies, in the case of the Peruvian population [5, 6], suggesting an isolation driven by the aforementioned geographical gap. The available data, in terms of total abundance estimates, indicates that the Peruvian/northern Chile population reaches up to 21,000 individuals, whereas the southern Chile population is approximately 65,000 individuals [7–9].

**Fig. 2:**
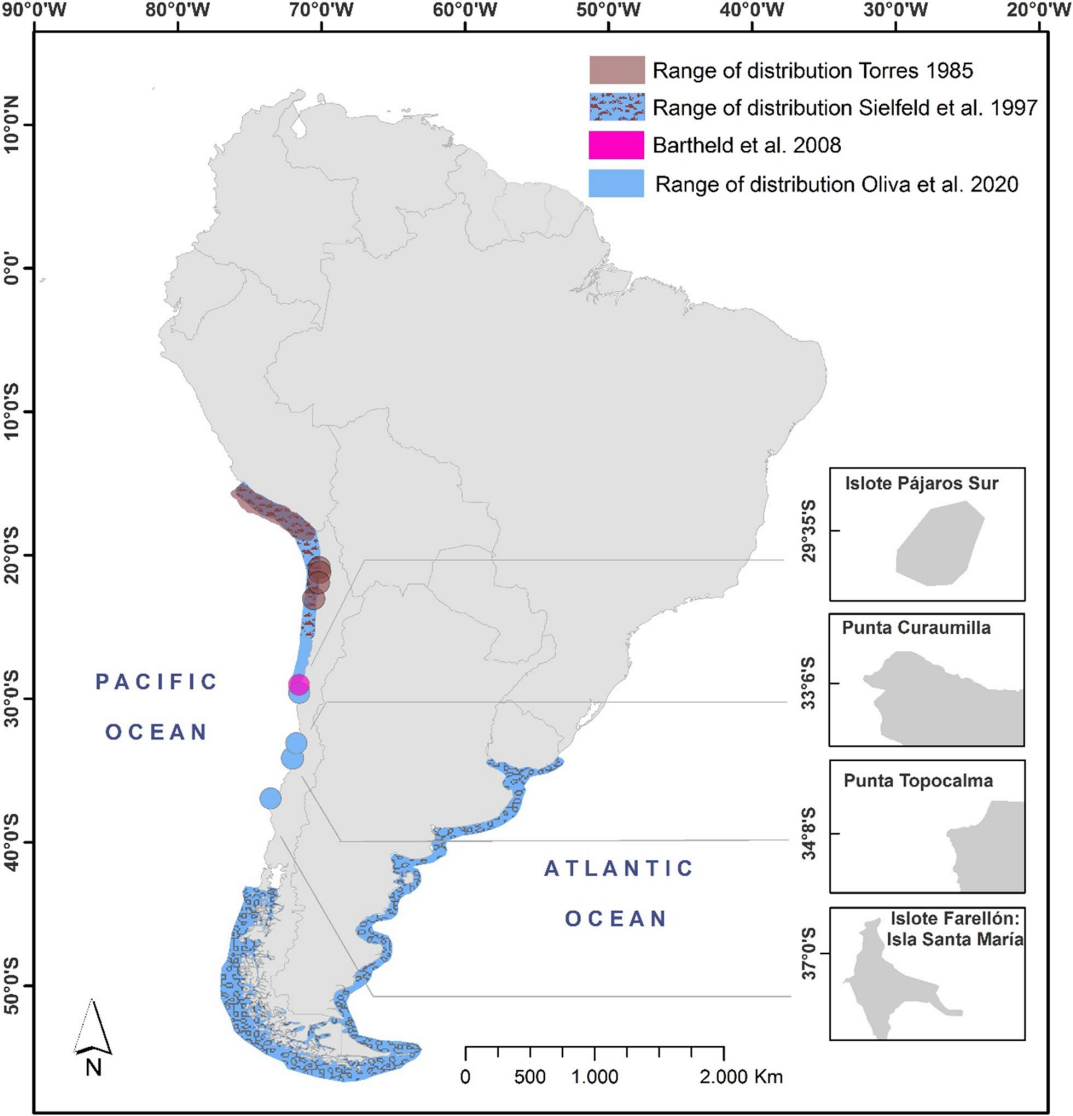
South American fur seal (*Arctocephalus australis*) range of distribution through time. Emphasis should be placed on the incipient colonization of northern Chile by some specimens [10], the establishment of reproductive colonies in northern Chile [8, 17] and the closing of the gap of distribution in central Chile ([22], this study). Map credit: Marlene Pizarro and Daniel Cárcamo. Image created using ArcGIS 10.8 and freely available to use

As a consequence of the strong ENSO event that occurred between 1982 and 1983 in Peru, a migration and displacement of SAFS individuals from the Peruvian colonies to northern Chile took place [10]. In February 1982, the first specimen of SAFS was registered in Roca Abtao (23° 05′ S), northern Chile. In a subsequent survey, 228 individuals were registered, with the largest numbers in Punta Paquica (n = 40) (21° 54′ S) and Roca Abtao (n = 93) (23° 05′ S), but also notably observed at Punta Comache (21° 11′ S) and Punta Patache (20° 51′ S) [10] (Fig. 2). The establishment of new SAFS colonies in northern Chile was presumably due to the animals’ need for food [10–16]. Moreover, while the abrupt decline in the effective population size of SAFS after the 1997–1998 ENSO event, in the year 1999 on the coast of Peru, was associated with greater mortality rates [15], it also coincided with a previously reported increase in the abundance of this species in northern Chile, with a total of 1600 individuals in the breeding season of 1996, with Punta Ballena (25° 49′ S) as the southernmost locality of the described distribution [3, 17] (Fig. 2). Additionally, in the summer season of 2007, Bartheld et al*.* [8] reported the presence of 17 SAFS in Isla Chañaral (29° 02′ S), which was the southernmost colony of the Peruvian subspecies (Fig. 2). Interestingly, we can also report that SAFS individuals had already entered the gap in its southernmost zone [4, 18, 19]. This suggests a colonization in both directions, with Isla Mocha (38° 25′ S) representing the northernmost colony of the southern Chile/Atlantic unit, and the aforementioned Isla Chañaral in northern Chile representing the Peruvian/northern Chile unit.

Monitoring the populations of this species within the gap of distribution is important in order to know its abundance and its population trend in Chile. This territory, apparently not inhabited by SAFS before, may now become a geographical area where individuals arrive, probably following the displacement of main prey items [12, 16]. Here, we examine the current abundance and distribution of SAFS on the central coast of Chile. An additional effort was made to trace past trends, by collecting and re-analyzing historical records. Thus, we were able to estimate an approximate date of arrival of individuals in mid-latitude Chilean waters.

## Main text

### Study area and survey planning

In central Chile, aerial censuses were performed during the austral summer of 2019 (17 February to 03 March) at a range between 29° 09′ S and 39° 24′ S, as well as during the austral spring (17 October) of 2019, at a range between 32° 12′ S and 34° 08′ S. Aerial censuses were performed by aircrafts (Piper PA-28R-180 Cherokee Arrow and Cessna R-172 Hawk XP II) at a velocity and height of about 90 kn and 250 masl, respectively [20]. In the austral summer (02 March) of 2020, a maritime census was performed at 33° 05′ S (Punta Curaumilla), using a 7 m fishing boat with an open desk (outboard engine, four strokes 40HP), and a velocity of about 5 kn. The summer survey data coincided with the post-breeding period of SAFS [21], since the surveys were intended to study the current distribution range and abundance of the sympatric otariid species, known as the South American sea lion (*Otaria byronia*) (SASL). Finally, an unmanned aerial census was carried out to observe and take close photographic records of individuals at Punta Curaumilla in (31 March) 2019, to confirm the presence of SAFS. The unmanned aerial census was carried out using a Drone (DJI Inspire I). All locations were georeferenced using Garmin GPS (Garmin Etrex 30× and Garmin Etrex Vista HCx), and photos were taken using digital cameras (CANON 7D, 6D Mark II and 40D with objectives 70–200 F/4 L IS, 100–400 F/4.5–5.6 IS and 28–135 F/3.5–5.6 IS). The collection of SAFS past records was based on the re-analysis of the graphic material obtained during scientific projects already concluded (see “Funding” section). Those were executed during the austral summer of years 1997 (13 January to 05 February-maritime census), 2007 (15 January to 16 February-aerial census) and 2015 (17 February to 14 March-aerial census) (see [22]). These censuses covered the same geographic area of our study and were also intended to study the abundance and distribution of the SASL.

### South American fur seal abundance and population structure

In the laboratory, a selection of photographs was put together to present the colonies without any of the photos registering an animal more than once. Based on secondary characteristics, individuals in the colonies were categorized in four functional age classes: Adult males, Adult females, Juveniles and Pups; when it was not possible to classify an individual, it was categorized as Indeterminate. Indeterminate individuals were proportionally assigned to Adult females and Juveniles, and not to Adult males or Pups since these two categories are easily recognizable by size and coloration [22]. We followed previous research conclusions in order to define a colony (> 25 individuals) and its subtypes, which can be classified as breeding colonies (reproductive structure and presence of > 15 pups) or non-breeding colonies (without reproductive structure and presence of < 15 pups) [22–24]. Censuses were performed by three independent trained observers using Adobe Photoshop CS6 Portable. One-way analysis of variance (ANOVA) was used to determine significant statistical differences between the total abundance, taking the sampling year as an independent variable into consideration. Furthermore, the test was executed with each observer as an independent variable. The assumptions of normality and homogeneity of variances were evaluated using the Shapiro–Wilk test and Levene’s test, respectively. For statistical comparisons that did not fulfill these requirements, we used the non-parametric Kruskal–Wallis test.

### New breeding and non-breeding colonies

We recorded the presence of fur seals in four different locations which are described as breeding colonies of the SASL: Islote Pájaros Sur (29° 34′ S), Punta Curaumilla (33° 05′ S), Punta Topocalma (34° 07′ S) and Isla Santa María: Islote Farellón (36° 57′ S) (Fig. 2). In the last three of the mentioned locations, the SAFS and the SASL colonies were spatially segregated. Interestingly, Islote Pájaros Sur seems to be a different ground from the other three, since the number of SAFS individuals counted (mean = 8 ± 1) does not meet the basic criteria to declare it a colony (it is probably an occasional haul-out site in the northernmost zone of the gap) (Fig. 2, Table 1).

**Table 1:** Aerial synoptic and partial censuses schedule and total fur seals sighted (*Arctocephalus australis*)

Location name	Date	Hour	Latitude	Longitude	M	F	J	P	Total	Type of colony
Islote Pájaros Sur	02.17.2019	15:27	29° 34ʹ S	71° 32ʹ W	4	4	0	0	8	Not applicable
Punta Curaumilla	03.03.2019	13:30	33° 05ʹ S	71° 44ʹ W	107	421	56	11	595	Non-breeding colony
Punta Topocalma	03.03.2019	16:57	34° 08ʹ S	72° 00ʹ W	25	15	0	6	46	Non-breeding colony
Islote Farellón	02.23.2019	15:17	36° 57ʹ S	73° 32ʹ W	82	145	0	19	246	Breeding colony
Total Summer 2019					218	585	56	36	895	
Punta Curaumilla	10.06.2019	09:29	33° 05ʹ S	71° 44ʹ W	20	23	3	0	46	Non-breeding colony
Punta Topocalma	10.06.2019	10:25	34° 08ʹ S	72° 00ʹ W	4	1	4	0	9	Non-breeding colony^a^
Total Spring 2019					24	24	7	0	55	
Punta Curaumilla	02.03.2020	08:09	33° 05ʹ S	71° 44ʹ W	220	457	44	23	744	Breeding colony^b^
Total Summer 2020					220	457	44	23	744	

During the austral summer of 2019, spring of 2019 and summer of 2020, along the distribution gap (29° 02′ S–43° 36′ S) for *Arctocephalus australis* in the southeast Pacific coast of Chile. Type of colony: Non-breeding (> 25 adult ind., < 15 pups), Breeding (> 25 adult ind., > 15 pups), Not applicable (< 25 adult ind.). Age classes: M: Adult males, F: Adult females, J: Juveniles, P: Pups

^a^Although this colony showed lower numbers of individuals in the spring aerial survey compared to the summer field sampling (does not meet the criteria stipulated by Grandi et al. [28]), we considered it a colony since it exhibited an increase in abundance and more than 25 adult individuals during the summer season of 2019

^b^Punta Curaumilla was listed as a non-breeding colony in the summer season of 2019 and as a breeding colony in 2020. We considered it a breeding colony in the text since it exhibited an increase in pup abundance over time

See hyperlinked to Additional file 1

In the Punta Curaumilla breeding colony, 46 ± 4 and 595 ± 7 SAFS were registered during the austral spring and summer of 2019, respectively, whereas 744 ± 5 individuals were registered during the summer of 2020, including 23 ± 2 pups (Figs. 1, 2, Table 1), the highest number ever counted in a single location in central Chile. No previous records exist for the same area.

In the non-breeding colony Punta Topocalma, totals of 1 ± 0, 16 ± 0 and 46 ± 3 SAFS were registered during the summer seasons of 2007, 2015 and 2019, respectively. During the spring of 2019, we registered 9 ± 1 individuals (Fig. 2, Table 1). In the SASL summer survey carried out during the breeding season of 1997, there are no records of fur seals, suggesting the arrival of individuals in the early 2000s, with Punta Topocalma standing out as the first non-breeding colony discovered in central Chile during the austral summer (2007).

Finally, in the Isla Santa María: Islote Farellón breeding colony, we registered the highest abundance of pups in a location (19 ± 0) for the summer synoptic census of 2019, with an overall abundance of 246 ± 6 fur seals (Fig. 2, Table 1). No records were found in the historical censuses.

The abundance pattern was significantly different (total number of individuals) across summer seasons between sampling years for the three main locations (Punta Curaumilla F_(4,10)_ = 31,804, p < 0.0001; Punta Topocalma H_(3)_ = 10.38, p < 0.016; Islote Farellón F_(3,8)_ = 6001, p < 0.0001). The analyses also showed that the abundance observed by each independent observer does not differ significantly (Punta Curaumilla F_(2,12)_ = 0, p = 1; Punta Topocalma H_(2)_ = 0.04, p = 0.981; Islote Farellón F_(2,9)_ = 0, p = 1).

After a reanalysis of the photographs for previous SASL censuses performed in central Chile, we detected only vagrant individuals, as shown in Punta Topocalma rookery. In the 2015 winter census (01 to 04 July), performed in the same geographical area, 43 SAFS individuals were found further south [4]. This census suggests the establishment of non-breeding colonies during winter only throughout the foraging season in the southernmost geographical area of the gap of distribution [4]. This location is only ~ 150 km away from the breeding colony found in this study during the summer survey of 2019, Isla Santa María: Islote Farellón.

The fur seal population in central Chile reached an overall abundance of 895 ± 4 individuals in the 2019 summer survey (from which 36 ± 1 were pups) (Table 1). These records are the most precise abundance data for fur seals in the area, since previous studies have no existing records, or the objectives were not focused on the identification of SAFS in the main area of study (Fig. 2).

### Implications for conservation

In the late eighteenth and early nineteenth centuries, there was an active marine mammal economic-linked capture activity, based on fur/oil-centered markets [25–27]. Even though there is limited information about SAFS captures in northern and central Chile [28], Torres et al. [7] indicate that during the middle of the nineteenth century, hundreds of ships were dedicated to the capture of fur seals in the Malvinas/Falkland, Los Estados, Mocha, Santa María, Juan Fernández and Desventuras islands. Due to this activity, the populations of SAFS were significantly reduced, and therefore it is highly likely that the gap of distribution on the coasts of Chile for this species was enhanced at that time.

The presence of pups suggests the establishment of new breeding grounds for SAFS in mainland Chile (~ 450 km south of Isla Chañaral and ~ 750 km north of Isla Guafo; main SAFS breeding grounds in Chile in the north and south, respectively) [22]. In the Atlantic Ocean at Fuegian Archipelago (Argentina) a recovery of the population abundance was also recently registered from 4157 in the 1990s to 9550 individuals in 2012. This recovery included a population shift with the settlement of new colonies and the change of the social structure of others. The studied population in Argentina showed a mean annual percentage of population increase of 6.1% and 4.1% for the overall and pup abundance, respectively [29]. When comparing this with the Chilean case, it is possible that our observations forecast this trend in the near future.

It is worth noticing that the Peruvian subspecies, listed as vulnerable, and the southern Chile/Atlantic unit listed as of least concern under The IUCN Red List of Threatened Species [2], will settle the gap without geographical and reproductive isolation, creating a challenge for conservation purposes.

## Limitations

The documented population growth and range expansion in central Chile is likely the result of a combination of two factors: a recolonization process after extensive hunting and subsequent El Niño phenomena. It is necessary to design a genetic analysis in order to identify the species/subspecies that is recolonizing the area. However, we expect to attain accurate growth rate estimates after meeting some specific recommendations, such as continuing to monitor the area during the breeding season and evaluating the detection efficiency of the different methodologies.

## Supplementary Information

**Additional file 1.** Counts of fur seals by independent observers in each census along the central coast of Chile.

## Data Availability

The raw data described in this Research note can be freely and openly accessed in Table 1 (hyperlinked to Additional file 1).

## Abbreviations

SAFS: South American fur seal
SASL: South American sea lion
M: Adult males
F: Adult females
J: Juveniles
P: Pups
I: Indeterminates
T: Total
x̄: Mean
SD: Standard deviation
CV: Coefficient of variation
