# Supplementary material for: Long-term monitoring for conservation: closing the distribution gap of Arctocephalus australis in central Chile
Source: BMC Res Notes. 2021 May 6;14:170. doi: 10.1186/s13104-021-05583-y (PMC8103573; doi:10.1186/s13104-021-05583-y)
Supplement: Supplementary file 1 — Additional file 1. Counts of fur seals by independent observers in each census along the central coast of Chile. [file 13104_2021_5583_MOESM1_ESM.docx]

**Additional file 1**

**1.** Counts of fur seals by independent observers in each survey location (austral summer 2019). Categorization classes: M: Adult males, F: Adult females, J: Juveniles, P: Pups, I: Indeterminates, T: Total. Statistical parameters: : Mean, SD: Standard Deviation; CV: Coefficient of Variation.

| **Location name** | **M** | | | **** | **F** | | | **** | **J** | | | **** | **P** | | | **** | **I** | | | **** | **T** | | | **** | **SD** | **CV** |
| --- | --- | --- | --- | --- | --- | --- | --- | --- | --- | --- | --- | --- | --- | --- | --- | --- | --- | --- | --- | --- | --- | --- | --- | --- | --- | --- |
| **Observers** | **1** | **2** | **3** |  | **1** | **2** | **3** |  | **1** | **2** | **3** |  | **1** | **2** | **3** |  | **1** | **2** | **3** |  | **1** | **2** | **3** |  |  |  |
| Islote Pájaros Sur | 4 | 4 | 3 | **4** | 4 | 4 | 4 | **4** | 0 | 0 | 0 | **0** | 0 | 0 | 0 | **0** | 0 | 0 | 0 | **0** | 8 | 8 | 7 | **8** | **0,6** | **7,5** |
| Punta Curaumilla | 102 | 119 | 100 | **107** | 410 | 414 | 405 | **410** | 53 | 49 | 60 | **54** | 10 | 10 | 12 | **11** | 14 | 10 | 16 | **13** | 589 | 602 | 593 | **595** | **6,7** | **1,1** |
| Punta Topocalma | 25 | 28 | 23 | **25** | 10 | 11 | 13 | **11** | 0 | 0 | 0 | **0** | 6 | 7 | 5 | **6** | 3 | 3 | 4 | **3** | 44 | 49 | 45 | **46** | **2,6** | **5,8** |
| Islote Farellón | 82 | 82 | 82 | **82** | 151 | 140 | 145 | **145** | 0 | 0 | 0 | **0** | 19 | 19 | 19 | **19** | 0 | 0 | 0 | **0** | 252 | 241 | 246 | **246** | **5,5** | **2,2** |

**2.** Counts of fur seals by independent observers in each survey location (austral winter-spring 2019). Categorization classes: M: Adult males, F: Adult females, J: Juveniles, P: Pups, I: Indeterminates, T: Total. Statistical parameters: : Mean, SD: Standard Deviation; CV: Coefficient of Variation.

| **Location name** | **M** | | | **** | **F** | | | **** | **J** | | | **** | **P** | | | **** | **I** | | | **** | **T** | | | **** | **SD** | **CV** |
| --- | --- | --- | --- | --- | --- | --- | --- | --- | --- | --- | --- | --- | --- | --- | --- | --- | --- | --- | --- | --- | --- | --- | --- | --- | --- | --- |
| **Observers** | **1** | **2** | **3** |  | **1** | **2** | **3** |  | **1** | **2** | **3** |  | **1** | **2** | **3** |  | **1** | **2** | **3** |  | **1** | **2** | **3** |  |  |  |
| Punta Curaumilla | 19 | 21 | 19 | **20** | 23 | 18 | 24 | **22** | 3 | 3 | 4 | **3** | 0 | 0 | 0 | **0** | 0 | 0 | 2 | **1** | 45 | 42 | 49 | **45** | **3,5** | **0,1** |
| Punta Topocalma | 5 | 4 | 4 | **4** | 0 | 0 | 1 | **0** | 4 | 4 | 4 | **4** | 0 | 0 | 0 | **0** | 0 | 0 | 1 | **0** | 9 | 8 | 10 | **9** | **1,0** | **0,1** |

**3.** Counts of fur seals by independent observers in Punta Curaumilla (austral summer 2020). Categorization classes: M: Adult males, F: Adult females, J: Juveniles, P: Pups, I: Indeterminates, T: Total. Statistical parameters: : Mean, SD: Standard Deviation; CV: Coefficient of Variation.

| **Location name** | **M** | | | **** | **F** | | | **** | **J** | | | **** | **P** | | | **** | **I** | | | **** | **T** | | | **** | **SD** | **CV** |
| --- | --- | --- | --- | --- | --- | --- | --- | --- | --- | --- | --- | --- | --- | --- | --- | --- | --- | --- | --- | --- | --- | --- | --- | --- | --- | --- |
| **Observers** | **1** | **2** | **3** |  | **1** | **2** | **3** |  | **1** | **2** | **3** |  | **1** | **2** | **3** |  | **1** | **2** | **3** |  | **1** | **2** | **3** |  |  |  |
| Punta Curaumilla | 223 | 219 | 217 | **220** | 409 | 394 | 399 | **401** | 37 | 37 | 41 | **38** | 22 | 23 | 25 | **23** | 57 | 66 | 62 | **62** | 748 | 739 | 744 | **744** | **4,5** | **0,0** |
